# Alzheimer’s Disease Neuropathological characterization of the dog brain and relationship to biofluid biomarkers and cognitive function

**DOI:** 10.21203/rs.3.rs-8994620/v1

**Published:** 2026-03-03

**Authors:** Abdullatif Alsulami, Marika Bogdani, Evan MacLean, C. Dirk Keene, Stephanie McGrath, Dog Aging Project Consortium, Caitlin S. Latimer, Julie A. Moreno

**Affiliations:** Colorado State University; University of Washington; University of Arizona; University of Washington; Colorado State University; Texas A&M University; University of Washington; Colorado State University

**Keywords:** Neurodegenerative diseases, Alzheimer’s disease (AD), Canine cognitive dysfunction (CCD), Thal’s staging, Braak staging, CERAD criteria, amyloid-beta (Aβ), neurofibrillary tangles (NFTs)

## Abstract

**Background:**

Alzheimer’s disease (AD) is a progressive neurodegenerative disorder and a major global health challenge affecting more than 55 million people worldwide. AD is clinically defined by progressive cognitive decline and neuropathologically characterized by the accumulation of amyloid-beta (Aβ) plaques and neurofibrillary tangles (NFTs). While transgenic rodent models have provided valuable mechanistic insights, they do not fully capture the spontaneous, age-related nature of human AD. In contrast, the companion dog develops naturally occurring age-associated cognitive decline and AD-like neuropathological features, including Aβ deposition and tau pathology, and behavioral impairments measurable by the validated cognitive scales, Canine Cognitive Dysfunction Rating (CCDR). However, the systematic application of human AD neuropathological criteria to canine brains has been limited.

**Objective:**

The objective of this study was to apply established human neuropathological criteria (Thal, Braak, and CERAD) to aged canine brains and examine relationships among neuropathology, cognitive status, and plasma biomarkers.

**Methods:**

Postmortem brain tissues from 24 client-owned senior dogs were evaluated using Thal phases for Aβ deposition, Braak-based regional assessment of tau pathology, and CERAD criteria for neuritic plaques. Neuropathological findings were integrated with antemortem owner-reported cognitive assessments and plasma biomarker measurements to evaluate the clinico-pathologic and biomarker associations.

**Results:**

Senior dogs exhibited Aβ deposition consistent with early to intermediate Thal phases, variable and regionally restricted tau pathology, and an absence of neuritic plaques. Quantitative analysis demonstrated greater Aβ burden in cognitively impaired dogs compared with cognitively intact dogs, while age, but not cognitive score, was strongly associated with regional Aβ burden. Further, plasma glial fibrillary acidic protein (GFAP) levels showed a significant positive correlation with Aβ plaque burden, whereas the other plasma biomarkers assessed did not.

**Conclusion:**

Senior dogs exhibit neuropathologic features consistent with early-stage AD-like pathology, characterized by Aβ deposition and limited tau pathology in the absence of neuritic plaques. These findings support the utility of the companion dog as a naturally aging model for investigating early ADrelated pathological processes and evaluating translational biomarkers during preclinical disease.

## Introduction

Alzheimer’s disease (AD), a progressive neurodegenerative disorder, affects over 55 million individuals worldwide. This number, and resulting social and economic burdens, is projected to triple by 2050 (Shin, 2022). AD is a clinical syndrome characterized by progressive cognitive decline and its defining neuropathologic features, collectively termed Alzheimer’s disease neuropathologic change (ADNC), include amyloid-β (Aβ) plaques and tau neurofibrillary tangles (NFTs). AD lacks effective disease-modifying therapies, underscoring the urgent need for innovative research approaches (Lei et al., 2021). Progress in the field has been hindered by limitations in transgenic rodent models, which, despite providing mechanistic insights, often rely on genetic modifications and fail to fully recapitulate the spontaneous, age-related, and multifactorial nature of human AD pathology (Drummond & Wisniewski, 2017). These limitations highlight the need for alternative animal models that better capture the complex interplay of genetic, environmental, and aging factors in AD.

The companion dog represents a distinctive and naturally occurring model of AD, exhibiting age-dependent cognitive decline and neuropathological features that overlap with key elements of human ADNC, including age-associated brain atrophy and the accumulation of Aβ plaques and NFTs (Abey et al., 2021; de Sousa et al., 2023; Schütt et al., 2016). In contrast to traditional rodent models, dogs share human environments, lifestyles, and daily exposures, providing a natural and translationally relevant context for studying the development and progression of AD-like pathology, but on a faster timescale. On average, a domestic dog lives approximately 10–12 years compared to an average human lifespan of 77 years in some populations (Galvani-Townsend et al., 2022; Montoya et al., 2023).

Importantly, dogs experience age-associated cognitive decline, termed canine cognitive dysfunction (CCD) syndrome, which typically affects dogs over eight years old and is associated with signs of dementia that are similar to those that occur in AD, including disorientation, altered social interactions, and functional decline (Prpar Mihevc & Majdič, 2019). Recent studies from the Dog Aging Project (dogagingproject.org) have advanced our understanding of CCD epidemiology by assessing signs of dementia in over fifteen-thousand dogs, revealing strong associations between CCD and factors such as age (Yarborough et al., 2022) and physical activity (Bray et al., 2023). Pathologically, Aβ accumulates progressively with age in dogs, both as extracellular plaques and soluble oligomers, mirroring AD-like pathology with changes in amyloid precursor protein (APP) processing that favors amyloidogenic cleavage (Pop et al., 2012). Tau pathology has generally been shown to be limited in canines. Recently, however, studies leveraging improved phospho-tau–specific reagents demonstrate regionally restricted tau pathology, particularly within limbic structures (Abey et al., 2021). Together, these observations suggest that dogs develop both clinical and neuropathological features relevant to AD. Despite this convergence, a critical gap remains: no rigorous studies have directly linked age-associated cognitive decline in dogs to the extent and regional distribution of AD-like neuropathology. This disconnect is due in part to the absence of a systematic, standardized framework for staging canine Aβ and tau pathology that is comparable to frameworks established for human AD.

To assess clinical behavioral changes in dogs, validated behavioral assessment tools such as the Canine Dementia Scale (CADES) and Cognitive Cognitive Dysfunction Rating (CCDR) scale are used to measure cognitive deficits in aged dogs and show striking similarities to human AD scoring systems like the Clinical Dementia Rating (CDR) (Bray et al., 2023; Madari et al., 2015; Olby et al., 2025; Salvin et al., 2011). Through evaluation of domains such as disorientation, social interaction, and daily functioning, these tools have revealed progressive cognitive decline in aging dogs, positioning them as a translational bridge between rodent models and human clinical studies (Chapagain et al., 2018; Haake et al., 2024). However, without corresponding standardized neuropathologic staging, the relationship between these clinical measures and underlying brain pathology remains poorly defined.

Although behavioral changes in aging dogs have been well described, comprehensive neuropathological mapping aligned with human AD frameworks (Montine et al., 2012) is lacking, hindering full validation of the canine model for AD. To address this gap, we applied established human AD neuropathological criteria, Thal phases for Aβ deposition, Braak staging for NFT distribution, and Consortium to Establish a Registry for Alzheimer’s Disease (CERAD) criteria for neuritic plaques assessment, to characterize AD-like pathology using client owned dogs (Montine et al., 2012). We further integrated plasma biomarkers used in human AD diagnosis that are also altered in CCD to examine their relationships with neuropathology and cognitive function (Alsulami et al., 2025; Fefer et al., 2022; Panek et al., 2020). By systematically aligning canine neuropathology with human AD staging and linking these findings to antemortem cognitive and biomarker assessments, this study aims to establish a unified clinico-pathologic framework for canine AD-like disease. Our findings demonstrate that AD-like pathology in aged dogs follows patterns similar to those observed in humans and that this pathology is associated with cognitive status. Through the application of standardized neuropathologic assessment, this work provides a structural foundation for validating the companion dog as a translational model of AD.

## Methods

### Recruitment and clinical assessment

Dogs were selectively recruited through multiple channels: social media and local news platforms, referrals from a network of primary care veterinarians identifying cases of suspected Canine Cognitive Dysfunction (CCD), and from the existing population of senior dogs evaluated by the Neurology Clinical Trials Service at the Colorado State University Veterinary Teaching Hospital, and from the Dog Aging Project’s Brain Health Study (McGrath et al., 2025). Informed consent was obtained from all pet owners prior to study enrollment. Clinical assessments included the Cognitive Cognitive Dysfunction Rating (CCDR) Scale, a validated survey instrument that measures signs of CCD. The inclusion criteria required absence of brain tumors or other neurological conditions that could affect cognition.

A cohort of the dogs were under the University of Washington IRB deemed that recruitment of dog owners for the Dog Aging Project, and the administration and content of the DAP Health and Life Experience Survey (HLES), are human subjects research that qualifies for Category 2 exempt status (IRB ID no. 5988, effective 10/30/2018). All study-related procedures involving privately owned dogs were approved by the Texas A&M University IACUC, under AUP 2021 − 0316 CAM (effective 12/14/2021). Details regarding age and cognitive scores of participating dogs are listed in Table 1. In this study, we used a modified classification threshold from ( ), dogs with a CCDR score > 40 were classified as CCD-positive (CCD+), and those with a score ≤ 40 were classified as CCD-negative (CCD−).

### Collection of plasma

Whole blood was collected, placed into EDTA blood collection tubes and centrifuged to separate plasma. Samples were stored at −80C until used for biomarker assays (see below). Plasma biomarker measurements were only available for a subset (n = 14) of the neuropathology cohort due to sample availability.

### Biofluid Biomarker Assay

The Quanterix SR-X platform was used to simultaneously measure Abeta 40 (Aβ40), Abeta 42 (Aβ42), Glial Fibrillary Acidic Protein (GFAP), and Neurofilament light chain (NF-L) in antemortem canine plasma samples (Simoa^®^ Neurology 4-Plex E Advantage Kit). Plasma samples were thawed, centrifuged, and diluted 4X in plasma diluent provided by the kit. Standards (in triplicate), and diluted samples (in duplicate) were loaded onto the plate and the assay performed according to the Simoa^®^ Neuro 4-Plex Advantage Plus kit for SRX instructions. Briefly, antibody conjugated beads and secondary detection antibodies were added to the plate and incubated overnight. After plate washing, activated RGP reagent was added, and the plate was incubated for 10 minutes. After washes and open air drying the plates were loaded onto the SRX. Quantification was performed using Quanterix SRX software version 1.2.0 using a 5-parameter sigmoidal curve generated from the standards. All sample aliquots were thawed only on the day of the assay, used once and discarded. Samples with responses above the dynamic range of the calibration curve (n = 14) were diluted 10X, rerun and reanalyzed.

### Neuropathology

#### Tissue collection and processing

Twenty-four canine brains were obtained postmortem after euthanasia and necropsy from privately owned dogs with owner consent. The brains were collected at the Veterinary Teaching Hospital at Colorado State University or the University of Washington and fixed in 10% neutral buffer formalin for at least 72 hours. Tissue blocks containing pre-frontal cortex, visual cortex, striatum, hippocampus, midbrain and cerebellum were processed using a Leica TP1020 Automatic Benchtop Tissue Processor, embedded in paraffin wax (Cancer Diagnostics, Cat #: EEPAR56), and sectioned on a Thermo Scientific HM 325–2 Manual Microtome at 5μm thickness and mounted on positively charged glass slides (Superfrost Plus, Cancer 232 Diagnostics, Cat #: 4951) for staining and analysis.

#### Immunohistochemistry

Immunohistochemical staining was performed to evaluate the pathologic hallmarks of ADNC. Aβ plaques were detected using Anti-β-Amyloid (1–16, 1:5000) on a Leica BondMax automated stainer with citrate antigen retrieval (PH 6.0). NFTs were evaluated using phospho-Tau (Thr181) Monoclonal Antibody (Thermo Fisher MN1050, 1:200) with manual staining. Briefly, paraffin-embedded sections were dewaxed in xylene and rehydrated through graded ethanols before antigen retrieval in 0.01M sodium citrate for 20 minutes at 95°C. Endoperoxides were removed by incubating sections in a 0.30% hydrogen peroxide and water solution for 30 minutes. To block non-specific labeling, sections were treated with 10% horse serum in Tris-A with 2% BSA (bovine serum albumin) and 2% Triton-X in Tris buffered saline (TBS). Sections were then incubated overnight at 4°C with primary antibodies diluted in Tris-A with 2% BSA. After washing with Tris-A with 2% BSA, sections were incubated for one hour with their corresponding biotinylated secondary antibodies (goat anti-rabbit or horse anti-mouse, Vector Labs) diluted in 10% horse serum in Tris-A with 2% BSA at 1:250. Slides were counterstained with hematoxylin, rinsed in bluing reagent, dehydrated through graded ethanols followed by xylene, and mounted with mounting media. Coverslips were applied using #1 cover glass.

To further evaluate classical AD neuropathological features, modified Bielschowsky silver stain was performed manually (Yamamoto & Hirano, 1986). Briefly, formalin fixed paraffin-embedded blocks were dewaxed in xylene, rehydrated through graded ethanols and incubated in fresh 20% silver nitrate for 20 minutes. Sections were then incubated in titrated ammoniacal silver solution and developed in a formalin-based developer. The reaction was monitored microscopically to optimize contrast. Slides were rinsed in distilled water extensively, treated with 2% sodium thiosulfate to remove unreduced silver, dehydrated through graded ethanols followed by xylene, and mounted with mounting media.

#### Histology Assessment

Neuropathological evaluation was performed by adapting National Institute on Aging-Alzheimer’s Association guidelines for AD neuropathology (Montine et al., 2012) to aging canines. All assessments were conducted blinded to clinical data, including animal age and cognitive scores. Histological assessment of Aβ deposits, NFTs, and neuritic plaques density was carried out on all dogs included in the study. An “ABC” score for neuropathological changes in canine brains was based on: (A) Aβ deposits, (B) NFTs, and (C) neuritic plaques. Brain sections analyzed for each assessment are shown in Supplemental Fig. 1.

#### Amyloid-β Pathology:

##### Thal Stage.

We evaluated six regions based on Thal’s 2002 staging criteria (Thal et al., 2002): Middle frontal gyrus and primary visual cortex (Thal Phase 1), hippocampus (Thal Phase 2), neostriatum (Thal Phase 3), midbrain (Thal Phase 4), and cerebellum (Thal Phase 5). For each region, we ensured that the tissue slices sampled comparable anatomical areas across all dogs. A brain region was considered positive for amyloid pathology if one or more plaques were identified in the tissue examined.

##### Semi-quantitative amyloid burden

Pathology assessment was performed using four categories

0 = no amyloid pathology,
1 = amyloid pathology covers less than half the tissue,
2 = amyloid pathology covers approximately half the tissue,
3 = amyloid pathology covers the entire tissue.

#### Phosphorylated Tau Pathology:

##### Braak Stage.

We assessed tau pathology for NFTs based on Braak staging criteria (Braak & Braak, 1991) in transentorhinal cortex (Braak stage I), CA1 (Braak stage II), subiculum (Braak stage III), entorhinal cortex (Braak stage IV), frontal cortex (Braak stage V), and primary visual cortex (Braak stage VI). An area was considered positive for pathology if any morphological NFTs were identified using an immunohistochemical stain for phospho-tau (Thr181).

#### Neuritic Plaque Density (CERAD Criteria):

We assessed neuritic plaque density using the CERAD criteria (Mirra et al., 1991) by evaluating the prefrontal cortex with the Bielschowsky silver stain. A neuritic plaque is characterized by the presence of dystrophic neurites that are typically highlighted on silver stain. The density is assessed as the number of plaques per 100x field.

### Digital Image Analysis

To quantify the burden of amyloid and phosphorylated tau (p-tau) pathology in the canine brain, histologic slides were digitized using an Aperio AT2 whole-slide scanner (Leica Biosystems; software version 102.0.7.5). All slides were scanned at 20× magnification using identical scanner settings (gain, brightness, and exposure) to minimize image-to-image variability. Regions of interest (ROIs) were manually delineated using HALO image analysis software (v3.4.2986; Indica Labs) and included hippocampal subfields and cortical gray matter.

For amyloid pathology, an area-based quantification approach was applied using the HALO Area Quantification module to calculate the percentage area occupied by positive staining within each ROI. Thresholding and masking parameters were optimized to accurately identify amyloid plaques while excluding nonspecific background staining and artifacts. For tau pathology, quantitative analysis was performed using Qupath software (version 0.6.0) (Bankhead et al., 2017; Nüesch et al., 2025). Briefly, a machine learning object classifier was trained to identify p-tau181 positive cells. Cell counts were normalized to area (cells/mm^2^).

### Statistical Analysis

Prior to conducting group comparisons, normality was assessed using the Shapiro-Wilk test, and Levene’s test was performed to check for homogeneity of variance. For group comparisons, two-sample t-tests were used where assumptions of normality and homogeneity were met and Mann-Whitney test when normality assumption was violated. Spearman’s correlation was computed to assess the association between CCDR scores, dog age in years, quantitive brain tissue Aβ and blood biomarker concentrations.

All statistical analyses were performed using R software (version 4.3.1) and GraphPad Prism (version 10.1.2), with statistical significance set at p < 0.05 for all analyses.

## Results

### Distribution of β-amyloid plaques

**Figure 1:**
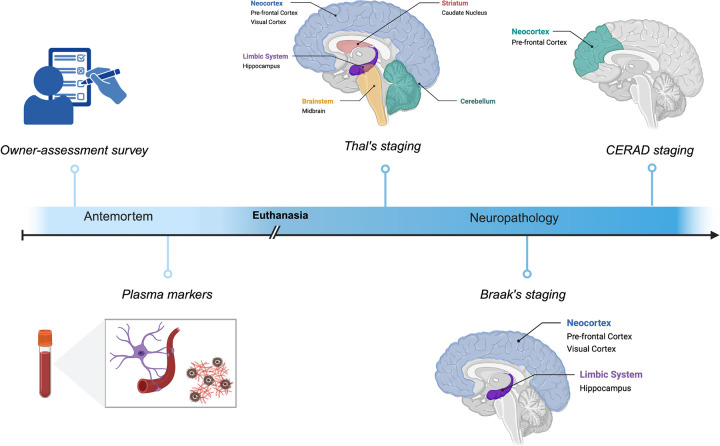
Study design and methodological overview. Schematic representation of the study workflow. Antemortem assessments included owner-based cognitive surveys and collection of plasma biofluid markers. Following euthanasia, postmortem neuropathological assessment was performed. Pathological evaluation included Thal’s amyloid phase, Braak’s neurofibrillary tangle stage, and CERAD scoring across multiple brain regions. Figure created with BioRender.

**Figure 2:**
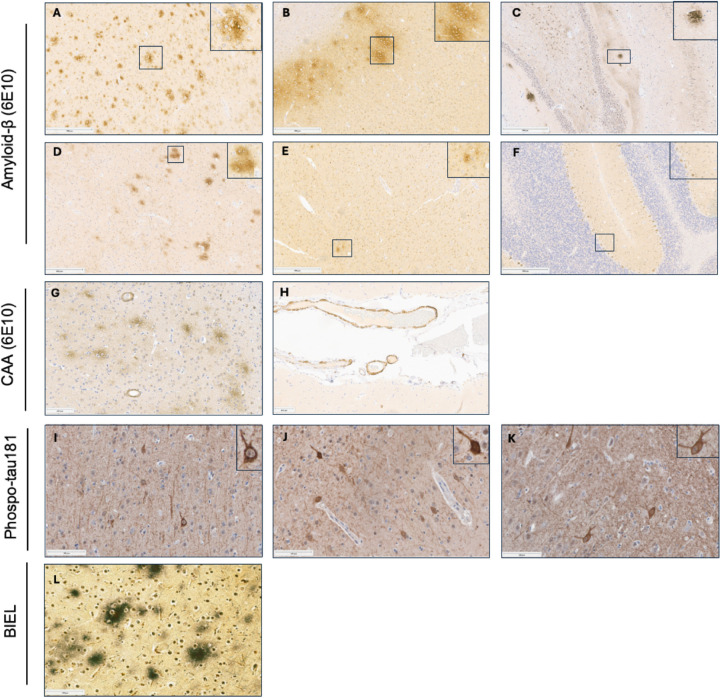
Senior canines show neuropathological characteristics similar to humans. Representative immunohistochemistry images showing Alzheimer’s disease-like pathology in canine brains. Panels (**A**–**F**) show β-amyloid plaques (6E10 antibody) in the prefrontal cortex (**A**), visual cortex (**B**), hippocampus (**C**), striatum (**D**), midbrain (**E**), and cerebellum (**F**). Panels G and H show cerebral amyloid angiopathy (CAA) in parenchymal vessels (**G**) and leptomeninges (**H**). Panels (**I**–**K**) show neurofibrillary tangles (NFTs) visualized with phospho-tau (p-tau181) antibody in the prefrontal cortex (**I**), visual cortex (**J**), and hippocampus (**K**), with insets highlighting positive cells. Panel (**L**) shows Bielschowsky silver staining in prefrontal cortex. Scale bars = 500 μm panels A-F, and 100 μm panels G-L.

**Figure 3:**
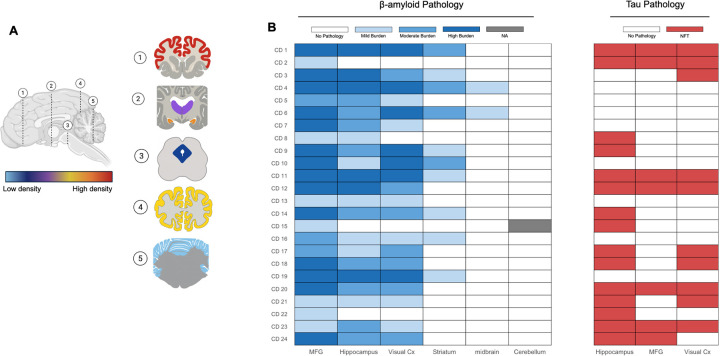
Regional distribution of β-amyloid and tau pathology in dogs. (**A**) Coronal brain sections illustrating the relative density of β-amyloid plaques, color-coded from absent pathology (light blue) to high pathology burden (red) based on semi-quantitative scoring. (**B**) Heatmap summarizing individual canine cases (CD1–24) for β-amyloid pathology (graded as no pathology, mild, moderate, or high burden) across neocortical and subcortical regions. Tau pathology is shown as the presence or absence of early neurofibrillary tangles (NFTs). Neocortical regions were predominantly affected by β-amyloid plaques, whereas hippocampus exhibited a higher burden of NFTs.

The extent of neuropathological changes in our cohort of aged dogs (eight years or older) was evaluated using criteria adapted from the NIA–AA guidelines for the neuropathological assessment of AD. Figure 1 shows the study pipeline using senior dogs. Figure 2 (Panels, A-F) show Thal’s phases for Aβ deposition. All dogs (100%) exhibited Aβ deposition in the neocortex. Aβ pathology was also observed in the hippocampus in 87.5% of dogs, the striatum in 37.5% of dogs, and the midbrain in 8% of dogs, while in no dogs did the cerebellum contain Aβ deposits (Fig. 3B).

### Distribution of tau neurofibrillary tangles

Neuropathologic examination of tau pathology was modeled after Braak regional staging principles using immunohistochemistry for phosphorylated tau (pTau181) (Fig. 2I–K). Tau pathology was less pervasive than amyloid, with 71% of animals exhibiting neurofibrillary tangle pathology. The hippocampus, particularly CA1, was most frequently involved (62.5%), with fewer animals showing extension into the frontal or primary visual cortices (46%) (Fig. 3B). Among animals with cortical involvement, most exhibited a hippocampus-to-neocortex progression consistent with Braak staging; however, 17% demonstrated atypical patterns, including neocortical involvement without hippocampal tangles or non-contiguous regional involvement.

### Neuritic plaque density

Using the criteria published by CERAD, we evaluated the brains for neuritic plaques. Despite widespread Aβ deposition detected by immunohistochemistry, no plaques meeting the morphologic criteria for neuritic plaques were identified by Bielschowsky silver staining. Accordingly, all animals received a CERAD score of absent (Fig. 2L).

### Alzheimer’s Disease Neuropathologic Change (ADNC)

To generate a comprehensive neuropathological score for each animal we combined the Thal, Braak and CERAD scores in accordance with the 2012 NIA-AA guidelines, adapted for canine neuropathology (Montine et al., 2012). Based on these criteria, the majority (80%) were classified as having LOW ADNC, while 16% met criteria for INTERMEDIATE ADNC; no animals met criteria for HIGH ADNC (Table 2). One animal exhibited Thal phase 3 amyloid deposition with neurofibrillary tangles restricted to the primary visual cortex. Because this pattern does not align with Braak staging assumptions, this case was excluded from ADNC classification.

### Cerebral amyloid angiopathy

Cerebral amyloid angiopathy (CAA) was observed in 9 dogs. Vascular-associated Aβ deposition was most prominent in posterior regions, particularly the occipital (visual) cortex, with a smaller subset showing involvement of both visual and frontal cortices.

### Regional burden of Aβ and tau pathology

Regional differences in amyloid burden were first assessed using a semi-quantitative scoring approach. The prefrontal cortex exhibited the highest plaque burden, followed by the hippocampus and visual cortex, while the striatum and midbrain showed minimal pathology (Fig. 3A). Across individual dogs, β-amyloid deposition was moderate to high in cortical and hippocampal regions, with considerable interindividual variability (Fig. 3B). Compared to amyloid pathology, tau pathology was less widespread and appeared more variable between cases (Fig. 3B).

### Quantitative analysis of β-amyloid and tau burden in CCD+ versus CCD− dogs

**Figure 4:**
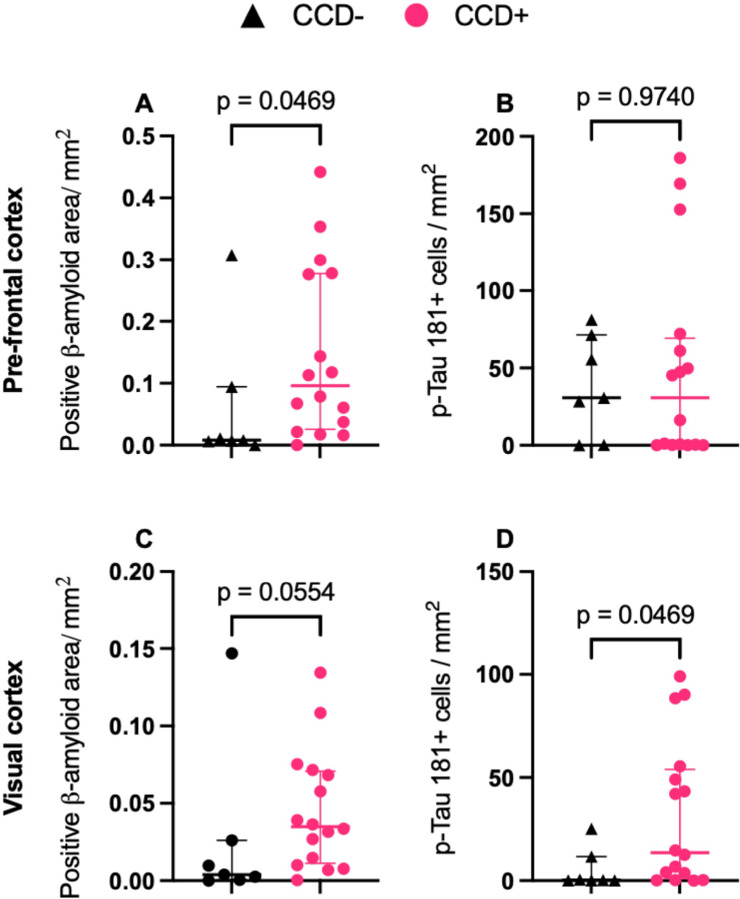
Region-specific increases in β-amyloid and P-tau181 pathology in cognitively impaired dogs. Quantitative assessment of β-amyloid pathology burden (**A**, **C**) and P-tau181 positive cells (**B**, **D**) in the pre-frontal and visual cortices of dogs with canine cognitive dysfunction (CCD+) and cognitively preserved dogs (CCD-). β-amyloid plaque burden was higher in the prefrontal cortex of CCD+ dogs (**A**; Hodges–Lehmann median difference 0.0582, 95% CI 0.00001 to 0.2592), whereas no difference was observed in the visual cortex (**C**; 0.0260, 95% CI −0.00018 to 0.0584). P-tau181–positive cells were increased in the visual cortex of CCD+ dogs (**D**; 12.42, 95% CI 0.0438 to 48.97), whereas no difference was observed in the pre-frontal cortex (**B**; −0.0051, 95% CI −31.45 to 49.61). Data are presented as median with interquartile range (IQR). Group comparisons were performed using Mann–Whitney tests.

Quantitative analysis revealed a significantly higher Aβ plaque burden in the prefrontal cortex of CCD+ dogs compared with CCD− dogs (Fig. 4A). No statistically significant difference was observed in the visual cortex between groups (Fig. 4C). In contrast, tau burden was significantly higher in the visual cortex of CCD+ dogs compared with CCD− dogs (Fig. 4D), and no significant differences were detected in the prefrontal cortex (Fig. 4B).

### Correlations of Neuropathology with Cognitive function and Age

Correlation analyses demonstrated a moderate association between age and Aβ plaque burden in the visual cortex (*r* = 0.57, p-value = 0.003), but weaker association in the prefrontal cortex (*r* = 0.35, p-value = 0.08). CCDR scores weak correlation with amyloid burden in both regions (Supplemental Fig. 3). Similarly, correlations between tau burden and age or CCDR scores were minimal across regions (Supplemental Fig. 4).

### Correlation of Blood Biomarkers with Amyloid Pathology

**Figure 5:**
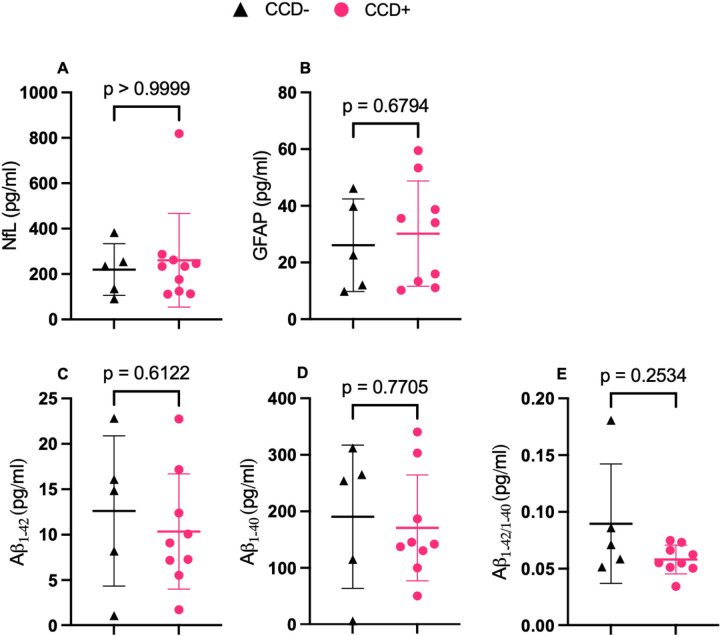
Plasma biomarker levels in aged dogs with and without cognitive dysfunction. Plasma concentrations of NfL (**A**), GFAP (**B**), Aβ1–42 (**C**), Aβ1–40 (**D**), and Aβ1–42/1–40 ratio (**E**) in dogs with canine cognitive dysfunction (CCD+) and cognitively preserved dogs (CCD-). No differences were observed between groups for any plasma biomarker (**A**; Hodges–Lehmann median difference 1.01, 95% CI −136.1 to 151.7; **B**; 1.29, 95% CI −23.86 to 26.63; **C**; −2.66, 95% CI −12.75 to 7.86; **D**; −14.77, 95% CI −166.34 to 131.57; **E**; −0.016, 95% CI −0.108 to 0.0039). No significant differences were detected. Data are presented as median with interquartile range (IQR). Group comparisons were performed using Mann–Whitney tests.

To determine if the underlying neuropathologic changes in the aged dog brains are associated with antemortem plasma biomarkers, we measured neurofilament light chain (Nf-L), Ab_1−40_, Ab_1−42_ and glial fibrillary acidic protein (GFAP). These biomarkers have previously been shown by our group and others to be associated with age, cognitive status, and neurodegeneration in dogs (Alsulami et al., 2025; Fefer et al., 2022; Panek et al., 2020). None of the plasma biomarker levels differed significantly between cognitively impaired (CCD+) and cognitively intact (CCD−) dogs for any of the analytes measured, including NfL, GFAP, Aβ_1–40_, Aβ_1–42_, or the Aβ_1–42_/Aβ_1–40_ ratio (Fig. 5). However, despite the absence of group-level differences, correlation analyses revealed a moderate positive association between plasma GFAP levels and regional β-amyloid plaque burden in both the prefrontal cortex and visual cortex (Supplemental Fig. 5C, D; *r* = 0.5, p-value = 0.04, for both regions). In contrast, plasma NfL levels and the Aβ_1–42_/Aβ_1–40_ ratio had minimal correlations with amyloid plaque burden across regions (Supplemental Fig. 4A, B, E, F) and plasma biomarkers demonstrated weak associations with tau burden(Supplemental Fig. 6).

## Discussion

### Aging dogs model early AD-like neuropathology

To determine whether and how the aging dog recapitulates key neuropathologic features of Alzheimer’s disease (AD) we applied National Institute on Aging–Alzheimer’s Association (NIA–AA) neuropathological criteria to dog brains. Using tools and approaches analogous to those used in human studies, we found, consistent with prior reports, that senior dogs develop β-amyloid (Aβ) plaques in an age-dependent manner that closely parallels early amyloid pathology in humans as described in Fig. 1. Notably, the neocortex appeared to be the earliest site of plaque deposition, as all dogs in our cohort exhibited at least some cortical Aβ pathology. This pattern is consistent with findings from human autopsy studies, in which approximately 86% of individuals exhibit cortical amyloid deposition (Thal et al., 2002). Similar to humans, amyloid pathology in dogs followed a hierarchical regional distribution with involvement of the hippocampus after the neocortex, followed by the neostriatum, and, in rare cases, the midbrain. Importantly, amyloid deposition did not extend beyond Thal Phase 2 in the majority of dogs, indicating that most aged dogs do not progress beyond the earliest stages of AD-like pathology (Fig. 2).

Consistent with this observation, no neuritic plaques, whichrepresent a more mature and pathogenic form of amyloid deposition, were identified in any canine brain examined, suggesting that aged dogs predominantly harbor early-stage amyloid pathology compared to humans.

Neurofibrillary tangles (NFTs) were also identified in limbic and, less frequently, neocortical regions, indicating that tau pathology is present in canine cognitive dysfunction (CCD); however tau pathology was less prevalent and less widespread than amyloid deposition and did not fully recapitulate the distribution observed in human AD. In this study, tau pathology was assessed using an antibody targeting phosphorylated tau at threonine 181 (pTau181) because the AT8 antibody, which is commonly used in human AD neuropathology, does not reliably label pathologic tau in the canine brain. Phosphorylation at Thr181 is considered an early tau modification in AD pathogenesis (Moloney et al., 2023). Together with the predominance of low Thal phases and the absence of neuritic plaques, these findings indicate that the dog brain exhibits neuropathology resembling the very earliest stages of AD, positioning dogs as a unique model for investigating early disease mechanisms.

### Amyloid and tau pathology show a hierarchical association in aging dogs

A major unresolved question in AD research concerns whether and how amyloid and tau pathology interact to drive disease progression. Our data provide some insight into this relationship in a natural aging model. All dogs with tau pathology also exhibited at least some degree of amyloid deposition, whereas not all dogs with amyloid pathology showed tau neurofibrillary tangles. Moreover, dogs with more severe amyloid pathology in the prefrontal cortex frequently exhibited greater tau burden in the hippocampus, and animals with hippocampal NFTs extending into neocortical regions typically had more advanced amyloid pathology (Thal phase ≥ 2) (Fig. 3). This pattern supports the hypothesis that amyloid pathology facilitates, but is not sufficient for, the development and spread of tau pathology (van der Kant et al., 2020).

### AD-like pathology precedes overt cognitive impairment in aging dogs

Quantitative analyses demonstrated that cognitively impaired dogs (CCD+) exhibited significantly greater Aβ plaque burden in the prefrontal cortex compared with cognitively intact (CCD−) dogs. In addition, CCD+ dogs showed significantly higher tau positive cells in the visual cortex, indicating that both Aβ and tau cortical pathologies are associated with cognitive impairment but may follow a region-specific pattern in the aging canine brain. However, some CCD− dogs displayed Aβ pathology consistent with Thal phase 2 and moderate to severe plaque burden. This may indicate that owner-reported behavioral assessments and CCDR scores may not fully capture subtle or preclinical cognitive changes, particularly in the earliest stages of disease. Alternatively, and consistent with observations in humans, some dogs may exhibit resilience to underlying neuropathology, resulting in a dissociation between cognitive performance and pathological burden (Chew et al., 2025; de Vries et al., 2024; Latimer et al., 2019).

Correlation analyses revealed that age was significantly associated with increased Aβ burden in the visual cortex (*r* = 0.57, p-value = 0.003), reinforcing age as a strong risk factor for pathology (Supplement Fig. 3). In contrast, CCDR scores were not significantly associated with neocortical Aβ burden (Supplement Fig. 3). Astrocytic GFAP plasma measurement showed significant positive correlation with Aβ burden in both regions (Supplemental Fig. 5B and E, *r* = 0.5, p-value = 0.05, for both regions). This finding parallels human studies demonstrating that increase in plasma GFAP levels reflect astrocytic activation secondary to Aβ pathology (Pereira et al., 2021). However, given the small sample size in our plasma cohort, this finding should be further investigated in a larger cohort of senior dogs. Tau pathology was not significantly associated with age, CCDR scores, or plasma biomarkers in any region (Supplemental Figs. 5 and 6). This observation is consistent with previous findings indicated that tau pathology in senior dogs is limited with variable distribution (Abey et al., 2021; Smolek et al., 2016; Wegiel et al., 1998; Yu et al., 2011). In contrast, studies in AD have demonstrated strong associations between tau pathology and cognitive decline (Brier et al., 2016; Hanseeuw et al., 2019). These results indicate that tau accumulation and distribution in senior dogs are more variable than amyloid pathology. This variability may suggest a more heterogeneous or more advanced stage of disease progression

Within this cohort, dogs with intermediate Thal phases (2–3) and higher tau pathology frequently exhibited neocortical involvement; however, marked heterogeneity was observed. Some animals showed substantial amyloid deposition with minimal tau pathology, whereas others exhibited tau pathology in the presence of limited amyloid burden. This variability suggests that additional pathogenic processes, including synucleinopathies, TDP-43 pathology, and vascular disease, may contribute to cognitive impairment and neuropathologic diversity in senior dogs, as found in human neurodegenerative disease (Kapasi et al., 2017; Latimer et al., 2019).

### Strengths and Limitations

A major strength of this study is the systematic application of established human AD neuropathological frameworks to a naturally aging canine cohort. By adapting Thal phases for Aβ deposition, Braak-based regional assessment of tau pathology, and CERAD criteria for neuritic plaques, this work provides the first integrated clinico-pathologic evaluation of AD-like changes in companion dogs using criteria directly comparable to those employed in human studies. Importantly, these comparative efforts were conducted in collabortion with human neuropathologists to ensure that staging approaches and pathological interpretations were alligned with similar standards used in AD research. The use of client-owned dogs further enhances translational relevance, as these animals experience heterogeneous genetic backgrounds, shared human environments, and natural aging trajectories that are not captured in traditional laboratory models. Integration of standardized neuropathologic assessment with validated owner-reported cognitive measures and antemortem plasma biomarkers represents an additional strength, enabling evaluation of relationships among pathology, cognition, and peripheral markers within the same individuals.

Several limitations should also be considered. First, the cross-sectional, postmortem design precludes inference regarding the temporal sequence of amyloid and tau pathology and their relationship to cognitive decline. Longitudinal studies incorporating serial cognitive assessments, biomarker measurements, and eventual neuropathologic evaluation will be essential to define disease trajectories in this model. Second, cognitive status was assessed using owner-reported instruments, which, while validated, may lack sensitivity to detect subtle or domain-specific impairments, particularly in preclinical disease stages (Hargrave et al., 2025). Third, tau pathology was assessed using a pTau181-specific antibody, as commonly used human antibodies such as AT8 do not reliably label dog tau; therefore, the full spectrum of tau pathology in dogs may not be captured. Additionally, this study was not powered to assess the influence of sex, breed, body size, or lifespan variability, all of which may contribute to heterogeneity in aging trajectories and neuropathologic burden as only 14 of our samples were analyzed for biofluid biomarkers.

Despite these limitations, the strengths of this approach, particularly the use of standardized human neuropathological criteria in a naturally aging, translationally relevant species, support the value of the companion dog as a model for investigating early AD-related pathological processes.

## Conclusions

Together, these findings support the validity of companion dogs as a naturally occurring model for AD-like pathology, particularly for investigating the earliest stages of disease. By integrating standardized human neuropathological frameworks with behavioral assessments and plasma biomarkers, our study bridges a critical gap between canine and human aging research. Future studies should expand the characterization of mixed neuropathological markers in dogs and further evaluate biomarker-pathology relationships using longitudinal and postmortem approaches. Such investigations will provide novel insights into the mechanisms that underlie CCD and may inform translational strategies relevant to both veterinary and human neurodegenerative disease.

## Supplementary Files

This is a list of supplementary files associated with this preprint. Click to download.

- SupplementalData.docx

## Figures and Tables

**Table 1:** Demographic information of dogs included in the study. Table listing individual dog identifiers (ID), age (years), and cognitive status scores, Canine Cognitive Dysfunction Rating (CCDR).

ID	Alt ID	Age	CCDR
CCD-3	CD 1	17.5	63
CCD-4	CD 2	13.2	51
CCD5	CD 3	14.8	48
CCD6	CD 4	13.3	42
CCD7	CD 5	14.7	46
CCD8	CD 6	17.5	55
CCD9	CD 7	13.5	45
CCD10	CD 8	14.5	39
CCD11	CD 9	16.5	61
CCD12	CD 10	18	63
CCD13	CD 11	14.8	41
CCD14	CD 12	10.7	44
CA514	CD 13	13.5	NA
CA516	CD 14	9	35
CA533	CD 15	10.1	37
CA541	CD 16	21	79
CA548	CD 17	13.5	38
CA569	CD 18	14	36
CA605	CD 19	15.5	44
CA703	CD 20	12.3	62
CA800	CD 21	12.4	45
CA350	CD 22	10.2	28
CA351	CD 23	13	51
CA352	CD 24	9	34

NA = data not available

**Table 2:** Distribution of dogs by Thal’s, Braak, and CERAD staging. Summary table showing the number of dogs assigned to Thal’s amyloid phases (A0–A3), Braak stages (B0–B3), and CERAD neuritic plaque scores (C0–C3). Most dogs exhibited intermediate Thal’s phases (A1–A2), variable Braak stages (B0–B3), and CERAD scores consistent with no neuritic plaques.

Thal’s staging		Braak’s staging		CERAD criteria	
	# of dogs		# of dogs		# of dogs
A0	0	B0	8	C0	24
A1	15	B1	0	C1	0
A2	9	B2	6	C2	0
A3	0	B3	9	C3	0

## Data Availability

All data supporting the findings of this study are available within the paper and its Supplementary Information.
